# Flower Feeding and Reproductive Timing in Spix's Night Monkeys (*Aotus vociferans*): Evidence From Arboreal Camera Traps

**DOI:** 10.1002/ece3.73918

**Published:** 2026-07-03

**Authors:** Claudia Viganò, Alessandro Mainardi, Pedro Alonso‐Alonso, Malika Gottstein, Víctor Romero, Richar Samaniego, Juan Pablo Uchuari, Katrin Heer

**Affiliations:** ^1^ Eva Mayr‐Stihl Professorship of Forest Genetics Albert‐Ludwigs Universität Freiburg Freiburg im Breisgau Germany; ^2^ Department of Animal Ecology and Tropical Biology, Biocenter University of Würzburg Würzburg Germany; ^3^ Museum für Naturkunde Leibniz Institute for Evolution and Biodiversity Science Berlin Germany; ^4^ Museo de Zoología Universidad Técnica Particular de Loja Loja Ecuador; ^5^ Naturaleza y Cultura Internacional Loja Ecuador; ^6^ Universidad Nacional de Loja Loja Ecuador

**Keywords:** canopy ecology, nocturnal mammals, phenology, plant–animal interactions, tropical rainforest

## Abstract

Tropical forest canopies harbor exceptional biodiversity, yet basic aspects of the ecology and reproductive biology of many canopy‐dwelling animal species remain poorly understood. This gap is particularly evident for small nocturnal arboreal mammals, which are challenging to study using conventional methods. Here, we used arboreal camera traps in a tropical mountain rainforest in Southern Ecuador to document flower visitors to the mass‐flowering tree *Handroanthus chrysanthus* . Over a 16‐month period, our recordings provided new information on the nocturnal and seasonal activity patterns of a group of Spix's night monkeys ( *Aotus vociferans* ). In this study, we report the first evidence of flower feeding on *H. chrysanthus* and suggest that seasonal activity patterns may be more closely aligned with the primate's reproductive timing than with peaks in floral resource availability. These findings demonstrate the power of arboreal camera traps for studying elusive canopy species and provide new insights into the behavior, feeding ecology, and reproductive biology of owl monkeys in the wild.

## Graphical abstract

This article provides new insights into the diet and reproductive biology of the Spix's night monkeys ( *Aotus vociferans* ). This evidence was collected using arboreal camera traps in a tropical mountain rainforest in Southern Ecuador, where documented information on this primate is rare.

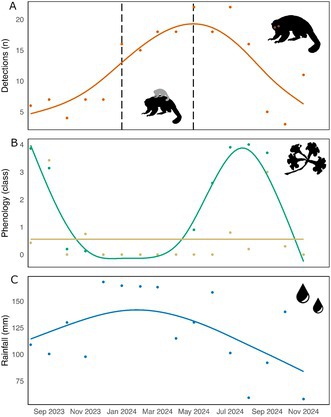

## 1 Introduction

Arboreal taxa comprise a substantial portion of rainforest mammal communities (Bowler et al. 2017; Haysom et al. 2023). In the Neotropics, primates are a prominent group of arboreal mammals. Among them, night monkeys—also known as owl monkeys (*Aotus* spp.)—are the only anthropoid primate genus recognized as strictly nocturnal (Link et al. 2023), although some species exhibit cathemeral behavior (Khimji and Donati 2014; Rotundo et al. 2000; Wright 1989). Their nocturnal and arboreal habits make direct observations difficult, contributing to the logistical challenges of studying these primates in the wild and resulting in relatively limited ecological information compared with other Neotropical primates (Fernandez‐Duque 2023; Montilla et al. 2021).

Night monkeys are relatively small‐bodied primates, weighing between 0.7 and 1.5 kg, and typically live in small groups composed of a reproductive pair and one or more immature individuals, with births usually consisting of a single infant (Fernandez‐Duque et al. 2020; Garcia de la Chica et al. 2023; Wright 1996). Males and females show minimal sexual dimorphism (Spence‐Aizenberg et al. 2018), and infant care is predominantly performed by the father (Dixson and Fleming 1981; Fernandez‐Duque et al. 2020; Wright 1990). The genus currently includes 11 recognized species, at least two of which occur in Ecuador (Defler and Bueno 2007; Gozalo and Elkins 2025).

Species of the genus *Aotus* occur from Panama to northern Argentina and inhabit a wide range of ecosystems, from lowland forests to elevations above 3000 m a.s.l. (Grow et al. 2014; Shanee et al. 2023). They typically occupy relatively small territories, generally ranging between 4 and 10 ha depending on the species and the habitat (Fernandez‐Duque et al. 2008; van der Heide et al. 2012; Wright 1989, 1996). Despite this broad distribution, relatively little is known about how their daily and seasonal activity patterns vary across different habitat types, particularly in tropical forests at low latitudes (Di Bitetti and Janson 2000). Because different *Aotus* species may respond differently to environmental conditions across habitats (Wright 1989; Montilla et al. 2021), describing their nocturnal and seasonal activity is an essential first step toward understanding their behavioral patterns in habitats that are so far understudied.

Night monkeys are generally considered omnivorous, feeding primarily on fruits and seeds but also occasionally consuming insects and flowers (van der Heide et al. 2023; Wright 1996). However, detailed information on their diet remains limited compared with other platyrrhines (Cooke and Klukkert 2023). This scarcity of information largely reflects the difficulty of observing nocturnal arboreal species in structurally complex tropical forests, where dense vegetation and tall canopy trees restrict visibility from the forest floor (Helenbrook et al. 2019; Heymann 2024).

Recent technological advancements have helped researchers overcome some of these challenges. Although tools such as thermal viewfinders have made observation from the ground possible during the night (Fernandez‐Duque et al. 2023), the limited visibility in structurally complex tropical forests still poses major challenges (Heymann 2024). Arboreal camera traps provide a promising non‐invasive alternative by enabling researchers to access forest strata that are otherwise difficult to monitor. By recording continuous data in the absence of researchers, camera traps additionally reduce the sampling effort (Moore et al. 2021) and allow longer monitoring periods. Nevertheless, detecting arboreal species remains more challenging than ground‐dwelling ones, as lower sampling effort on the ground can lead to a faster saturation of species accumulation curves (Haysom et al. 2021).

Detection rates for arboreal species may increase when cameras are placed in trees that provide attractive resources. Mass flowering events in tropical forests are typically short‐lived but highly conspicuous, producing abundant and energy‐rich resources that can attract a wide range of visitors (Barros 2001; Gentry 1992). These resource pulses may temporarily concentrate the activity of opportunistic feeders such as *Aotus* species around particular trees, potentially increasing their detectability (Garber 1988; Heymann 2011; van der Heide et al. 2023). Beyond their energetic value, flowers may also provide nutrients or secondary compounds that influence physiological processes, including reproduction. For example, female mantled howler monkeys ( *Alouatta palliata mexicana* ) increase flower consumption during lactation to regulate tannin intake, suggesting a link between diet composition and reproductive timing (Gisbrecht et al. 2025). However, whether similar relationships occur in *Aotus* species remains largely unexplored.

In this study, we observed a group of Spix's night monkeys ( *Aotus vociferans* ) in a tropical mountain rainforest in southern Ecuador, providing new insights into their feeding ecology and aspects of their reproductive biology in the wild. Using tree‐climbing techniques, we placed camera traps in the canopy of a tree species belonging to the Bignoniaceae family: *Handroanthus chrysanthus* (Jacq.) S.O.Grose. This species was selected because it produces large, conspicuous yellow flowers in large quantities (Bendix et al. 2006; Cueva‐Ortiz et al. 2006). The peak flowering period usually occurs between July and October and lasts approximately one month, with the potential of attracting many animal taxa during that time period (Borrero 1972; Gentry 1992). Night monkeys were already reported to feed on the flowers of another member of the same plant genus: *Handroanthus heptaphyllus* (van der Heide et al. 2023). Nevertheless, while florivory has been observed in previous studies of *Aotus*' diet (Montilla et al. 2021), the consumption of the flowers of *H. chrysanthus* had not been previously recorded.

Because the mass flowering of *H. chrysanthus* may temporarily concentrate the activity of *A. vociferans* around these trees, in this work we investigated the ecological interactions between these primates and this seasonal resource pulse. Specifically, we as follows:

- characterized seasonal activity patterns of the *A. vociferans* group in our study site, to determine whether activity peaks occurred during a specific period of the year. Given that cameras operated exclusively at night, we also described the nocturnal distribution of detections to verify that our sampling captured biologically realistic activity patterns consistent with the expected bimodal nocturnal activity pattern described for *Aotus* (Link et al. 2023);
- documented the interactions between *A. vociferans* and *H. chrysanthus* to evaluate the extent to which these primates were attracted to its flowers;
- assessed whether variation in *A. vociferans* activity throughout the year coincided with the short flowering period of *H. chrysanthus* , or was instead better explained by seasonal aspects linked to the night monkeys' life cycle, particularly the reproductive cycle (Bronson 1989).

## 2 Methods

### 2.1 Study Site

**FIGURE 1:**
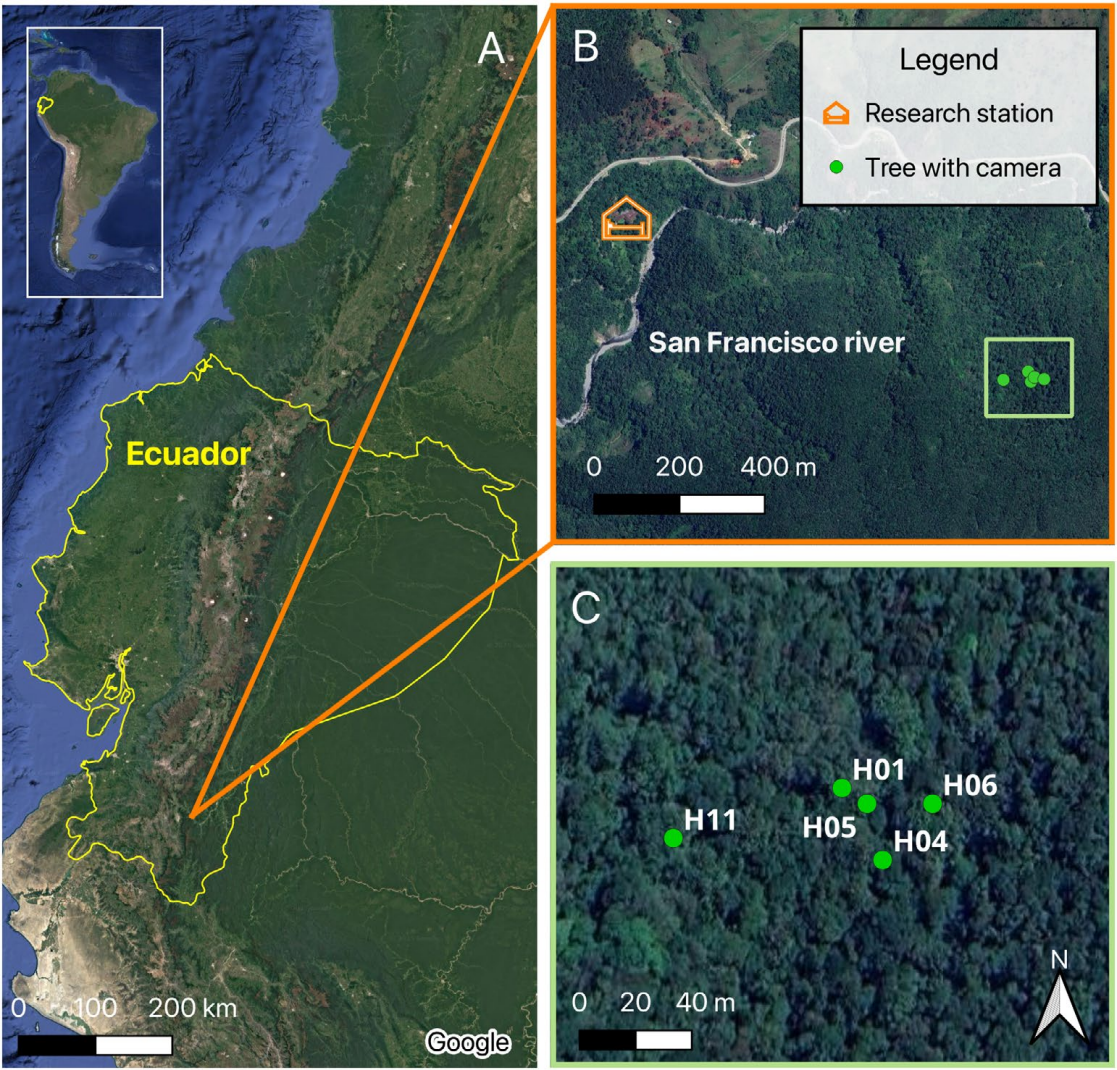
Location of the research site in southern Ecuador (A). The research station (B), situated near the San Francisco River, provided daily access to the study plot where five *Handroanthus chrysanthus* trees equipped with camera traps (B, C) were monitored.

We collected the data in an evergreen mountain rainforest (Cueva‐Ortiz et al. 2006) located at an elevation of approximately 2000 m a.s.l at the Estación Científica San Francisco (ECSF, 3°58′27.6″ S, 79°04′13.4″ W), located within the Reserva Biológica San Francisco (RBSF, 3°58′27.6″ S, 79°04′13.4″ W). The RBSF extends for 1000 ha between 1800 and 3150 m a.s.l. (Bussmann 2003) on the eastern Andean slope of southern Ecuador (Figure 1), near the border of the Podocarpus National Park. The area is characterized by a tropical perhumid climate (annual rainfall average of 2067 mm) with a wet season from April to July (above 200 mm per month), followed by a drier period from September to December (below 150 mm per month) (Cueva‐Ortiz et al. 2006). The study area harbors high levels of plant and animal diversity (Beck et al. 2008) and the vegetation is dominated by Lauraceae, Melastomataceae, and Rubiaceae (Cueva‐Ortiz et al. 2006). On a floristic level it can be classified as *Ocotea‐Nectandra* forest (Bussmann 2003).

### 2.2 Data Collection

From August 2023 to November 2024, we installed five camera traps (Reconyx HyperFire 2, Reconyx, US) in the canopy of five *Handroanthus chrysanthus* trees (Figure 1). Tree height ranged from 25 to 32 m (mean ± SD: 28.4 ± 2.6 m; CV = 9.2%), and diameter at breast height (DBH) ranged from 44.9 to 70.38 cm (59.2 ± 9.7 cm; CV = 16.3%). (Table S1).

*Handroanthus chrysanthus* is a widespread tree species, covering a broad latitudinal and elevational range (from sea level to ~2000 m a.s.l.) and is economically important for timber production (Cueva‐Agila et al. 2021). Little ecological research has examined the interactions between this plant and the animals that exploit the seasonally abundant trophic resources it provides (e.g., flowers). We firstly selected these trees as part of a parallel phenological monitoring study conducted within our project. The abundance of flower buds and open flowers on these trees was visually recorded twice per month using phenological classes (Table 1) based on a standardized monitoring protocol (Denny et al. 2014).

**TABLE 1:** Phenological intensity classes (Class) and respective assigned values, in terms of quantity (*Q*) and percentage (*P*), of each flowering phenological phase: Flower buds and flower production.

Class	*Q* (Flower buds)	*P* (Flowers)
1	< 3	< 5%
2	3–10	6%–25%
3	11–100	26%–50%
4	101–1000	51%–75%
5	1001–10,000	76%–94%
6	> 10,000	95%–100%

*Note:* The percentage refers to the total number of open flowers compared to the total number of flower buds and flowers present on the trees. Modified from the classes defined by Denny et al. (2014).

Additionally, to collect information on the animal species interacting with *H. chrysanthus* , we installed camera traps in the canopy of these trees. The cameras were mounted between 21 and 25 m (Table S1) above the forest floor. They were motion‐activated from 18:00 to 06:30 to capture both nocturnal and crepuscular activity. Using an infrared sensor, they recorded 30‐s videos in low‐light conditions. We activated the “no delay” option between triggers and before re‐triggering to maximize capture rates. For camera placement, we selected individual adult trees based on the following criteria: (I) they possessed large, stable, and accessible branches surrounded by similarly structured branches bearing floral resources (flowers or buds); and (II) they were already part of the phenological monitoring scheme, enabling us to explore potential links between tree phenology and animal activity. After initial adjustments to camera position between August and November 2023, the cameras were maintained at the same position for the remainder of the study period (Figure S1). The average distance between trees observed with cameras was 44.37 m (min. 10.5 m–max. 93 m; Table S2). The five focal trees defined the vertices of a minimum convex polygon (MCP) of approximately 0.12 ha, representing the spatial extent of the study area. We calculated the MCP using the *sf* package in R version 4.5.1 (R Core Team 2025), after projecting the tree geographic coordinates (Table S1) to UTM Zone 17S.

The cameras were checked once per month while performing phenological monitoring on the trees. Both the cameras' installation and the phenological monitoring, which recorded the presence and abundance of flower buds and flowers (Table 1), were performed by climbing on each tree using single‐rope techniques.

### 2.3 Taxonomy

Based on the examination of a subadult female found dead at the study site, we provisionally identify the night monkeys population at the research station Estación Científica San Francisco (ECSF) as *Aotus vociferans* (Spix, 1823). The specimen was found and deposited at the Zoology Museum of the Universidad Técnica Particular de Loja, Ecuador (MUTPL M‐1303) by museum personnel independently of this study, and no zoological material was collected or handled by the authors for this purpose.

The identification remains nevertheless uncertain, as the morphological traits traditionally used to delimit *Aotus* species, especially pelage coloration and hair length, are highly variable and often non‐diagnostic across the genus (Fernandez‐Duque et al. 2013). Furthermore, this locality lies at the potential convergence of the distributional ranges of *A. lemurinus, A. vociferans*, and *A. nancymae* (Martins‐Junior et al. 2022). Thus, molecular verification (using mtDNA and nuclear markers) will be required to confirm the taxonomic identity of the ECSF population. Until such evidence is available, we retain the assignment of the observed group to *A. vociferans* as a working hypothesis.

### 2.4 Identification of Flower‐Feeding Visits and Independent Detection Events

To identify flower visitors on *H. chrysanthus* and characterize their behavior, we analyzed all camera‐trap videos using the TimeLapse software (Greenberg et al. 2019). All observations of species identity and behavior were conducted by the same person (CV) to ensure consistency. For the present study, we focused on videos in which *A. vociferans* was detected.

To ensure independence among detections (i.e., among individual video records of *A. vociferans* ), we applied a 30‐min temporal threshold (Ridout and Linkie 2009; Haysom et al. 2021). Consecutive videos of *A. vociferans* recorded at the same tree on the same night were considered part of the same detection event if they occurred within 30 min of the previous detection. A new detection was thus considered independent only when at least 30 min had elapsed since the most recent detection. When multiple videos occurred within the same event (i.e., because they were all recorded less than 30 min apart), we retained a single record corresponding to the maximum number of individuals observed in any 30‐s video within that period. This approach ensured that a single detection event was not artificially divided into multiple detections and reduced the risk of underrepresenting the number of individuals present simultaneously on the tree. At the same time, this procedure avoided inflating detection counts due to repeated recordings of the same individual or individuals within short time intervals.

#### 2.4.1 *Aotus vociferans'* Behavior, Minimum Group Size and Infant Observations

Based on the most frequently observed behaviors *of A. vociferans*, we categorized them into an ethogram (Table 2). We were particularly interested in detection events that provided information on diet and direct interactions with the *H. chrysanthus* trees. When we recorded the observed individuals feeding on the plant's flower buds or flowers, we defined this as “florivory”, while we assigned herbivory, insectivory, or frugivory events to the more general “foraging” category. This was done because we were mainly interested in the attraction of *A. vociferans* to flower structures, since *H. chrysanthus* does not produce fleshy fruits, but a dry, dehiscent capsule (Grose and Olmstead 2007) that was not consumed by *A. vociferans* . We classified the videos as “locomotion” or “inspecting” if *A. vociferans* was not observed eating but was just observed moving or inspecting other plant structures (e.g., branches), respectively. In videos showing multiple behaviors, we selected only a single behavioral category, giving priority to “foraging” or “florivory” events over the others. We then calculated the relative frequency of each behavior based on the total number of *A. vociferans'* detections. We additionally documented the presence and carrying position of the infant and estimated the minimum group size by counting the number of individuals recorded in a video.

**TABLE 2:** Ethogram reporting the behavior (first column) assigned to the most frequently observed actions, and the definition (second column) of the behavior.

Behavior	Definition
Florivory	Grabbing the flower bud or flower of *H. chrysanthus* and approaching the base of it to the mouth, sucking the nectar, and tossing the corolla away (i.e., florivory or nectarivory).
Foraging	Removing an insect from the plant's leaf and eating it (insectivory). Removing a piece of a fruit (frugivory) or leaf (herbivory) and eating it.
Inspection	Approaching with the face, or grabbing with the hand and observing from closer, a tree organ (leaf, bud, flower…) or the epiphytes on the branches.
Locomotion	Walking or jumping in front of the camera.
Other	None of the behaviors described above (e.g., interaction with other individuals; ND).

### 2.5 Data Analysis

#### 2.5.1 Nocturnal and Seasonal Activity Pattern

We used the total number of independent detections of *A. vociferans* obtained from the camera traps' recordings to estimate its nocturnal and seasonal activity pattern. Because our camera trap data were event‐based (i.e., detections are not continuous), we used the time of each independent detection as a proxy for activity (Rowcliffe et al. 2014). We performed all analyses using R version 4.5.1 (R Core Team 2025). To account for the circular nature of time across daily cycles, we first processed detection timestamps, converting them to radians using the *lubridate* (Grolemund and Wickham 2011) and *dplyr* (Wickham et al. 2023) packages. We then estimated nocturnal activity patterns using the *overlap* (Meredith et al. 2024) package, which implements non‐parametric kernel density estimation on circular time data. The resulting density curves depict *A. vociferans'* activity pattern over a 24‐h cycle, with the caveat that activity was only recorded from 18:00 to 06:30.

**FIGURE 2:**
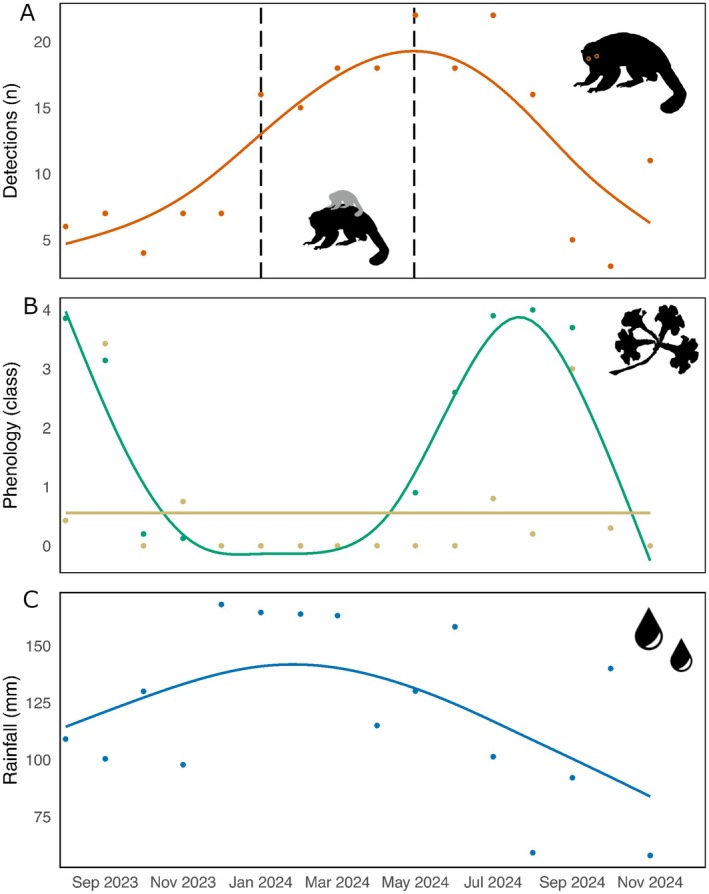
Seasonal patterns of *Aotus vociferans* activity and covariates. (A) Total monthly detections (*n* = 195) of *A. vociferans* with the time‐window in which infants were detected delimited by dashed black lines (first appearance: January 2024; last: April 2024). (B) Flowering phenology of flower buds (green) and open flowers (yellow), expressed as phenological class values. (C) Monthly rainfall calculated as total precipitation (mm). Curves were fitted using generalized additive model (GAM) smoothing functions applied to monthly *A. vociferans* independent detections, flowering phenology average class scores of flower buds and open flowers, and monthly precipitation.

Finally, we aggregated all independent *A. vociferans* detections per month across all trees over the entire observation period to characterize the seasonal activity pattern of the *A. vociferans* group. To visualize seasonal patterns (Figure 2), we applied smoothing curves using the geom_smooth() function in the R package *ggplot2* (Wickham 2016), specifying a generalized additive model (method = “gam”) to monthly *A. vociferans* independent detections, flowering phenology (flower buds, FB; open flowers, OF), and precipitation. For graphical purposes, we aggregated precipitation at the monthly level, based on data from an on‐site climatic station (Bendix et al. 2025), and for flowering phenology, we calculated the monthly mean phenological class across the monitored tree for both flower buds (FB) and open flowers (OF) (Table 1). Because we recorded FB and OF as ordinal phenological classes, the plotted phenology values represent average class scores. In addition, we produced descriptive statistical summaries of all variables (Table S5).

#### 2.5.2 *A. vociferans* Activity Pattern in the Context of Flower Availability and Seasonality Effects

To quantitatively assess whether temporal seasonality, or one of the measured predictors (flowering phenology and precipitation), drives the activity pattern of *A. vociferans* during the 16‐month study period, we fitted negative‐binomial generalized linear mixed models (NB‐GLMMs) using the package *glmmTMB* (McGillycuddy et al. 2025), with a log link function.

We defined the response variable as the number of independent detections of *A. vociferans* per tree (*n* = 5) in a given month (*n* = 16). Although the sampled trees were located in close proximity and may not be fully spatially independent, we did not explicitly model spatial correlation among trees given the small number of sampling units (*n* = 5). Instead, we included the tree as a random intercept to account for repeated observations and differences in detection rates among *H. chrysanthus* individuals.

We selected a negative‐binomial error distribution because the raw counts of *A. vociferans* ' independent detections were over‐dispersed (variance/mean = 4.73), thus violating the assumptions of a Poisson distribution. Our fixed effects comprised:

– Flowering phenology as a proxy for resources availability: we included flower buds as a numeric ordinal predictor corresponding to the monthly mean phenological class in Table 1, calculated for each monitored tree. Because these classes represent ordinal abundance scores of flower buds, higher classes indicate greater resource availability, but differences between classes should not be interpreted as equal quantitative increases. To account for potential differences in floral resource availability associated with tree size, we weighted the monthly mean flower bud class scores of each monitored tree by its DBH. Since we did not directly quantify the relationship between tree size and flower abundance, we used DBH as a proxy and log‐transformed it before inclusion in the model. This provided a more conservative weighting approach and avoided assuming a strictly linear relationship between tree size and flower abundance. We did not include open flowers (OF) in the models because their mean class values were close to zero for most months. The reduced flowering intensity in September 2024, compared to the previous years, was probably due to the drought resulting from the El Niño Southern Oscillation (ENSO) phenomenon that took place in 2023–24 (NASA 2024);
– Precipitation as a proxy for climatic seasonality: we included total monthly precipitation (PCP) recorded at the on‐site climatic station (Bendix et al. 2025) to capture potential effects of rainfall variability under the seasonal regime of our study area, which may influence primate activity patterns or food availability;
– Annual cyclicity as a proxy for time of the year: we included two circular terms, sinM (sin (2*π* calendar month/12)) and cosM (cos (2*π* calendar month/12)), to represent the continuity of the annual cycle (Bush et al. 2017). Because seasonal patterns repeat annually, representing time as circular variables avoids artificial breaks between December and January. Both sine and cosine terms can be included in the model (Stolwijk et al. 1999) to determine how strong the seasonal variation is (i.e., the magnitude of change between the lowest and highest levels of activity) and when during the year the peak in activity occurs.

We explored pairwise associations among the selected predictors (DBH‐weighted flower buds, PCP, sinM and cosM) using Spearman rank correlations and assessed multicollinearity using variance inflation factors (VIF). VIF values below 5 were considered indicative of acceptable levels of collinearity (James et al. 2013). Given the seasonal nature of flowering phenology and precipitation, we expected some degree of association among predictors. Therefore, these diagnostics were used to evaluate potential redundancy among predictors prior to model interpretation.

We then compared every possible combination of these four fixed effects using the corrected Akaike Information Criterion (AICc) via the dredge function in the *MuMIn* package (Bartoń 2025), to identify the combination of predictors best explaining the variation in *A. vociferans* detections. We selected AICc over the standard AIC since it provides a better model selection metric adjusting for small sample sizes. Because several models received similar support, we calculated model‐averaged parameter estimates across the set of models with ΔAICc ≤ 2 using Akaike weights (Burnham and Anderson 2002).

To ensure robust inference, we conducted standard post‐estimation diagnostics on the top‐ranked model using the *DHARMa* package (Hartig 2024), as simulation‐based residual checks require a single fitted model object. Residual diagnostics were used to verify key model assumptions, including dispersion, zero inflation, and residual uniformity. To improve interpretability, we expressed log‐scale coefficients as rate ratios with Wald 95% confidence intervals.

## 3 Results

### 3.1 Video Analysis

The recording period started at the beginning of August 2023 and ended at the end of November 2024 (Table S3) for a total of 478 nights, and the total sampling effort was 2183 camera‐trap nights (CTNs). The total number of videos recorded was 4092. Of those, 7.28% (*n* = 298) recorded *A. vociferans* , whereas 30.67% (*n* = 1255) recorded different animal species not considered in this study. The remaining videos were false triggers caused by wind. Only the records with independent detections (i.e., consecutive videos at the same tree separated by at least 30 min) of the study species (*n* = 195) were retained for the analysis.

### 3.2 Behavior and Group Structure

**FIGURE 3:**
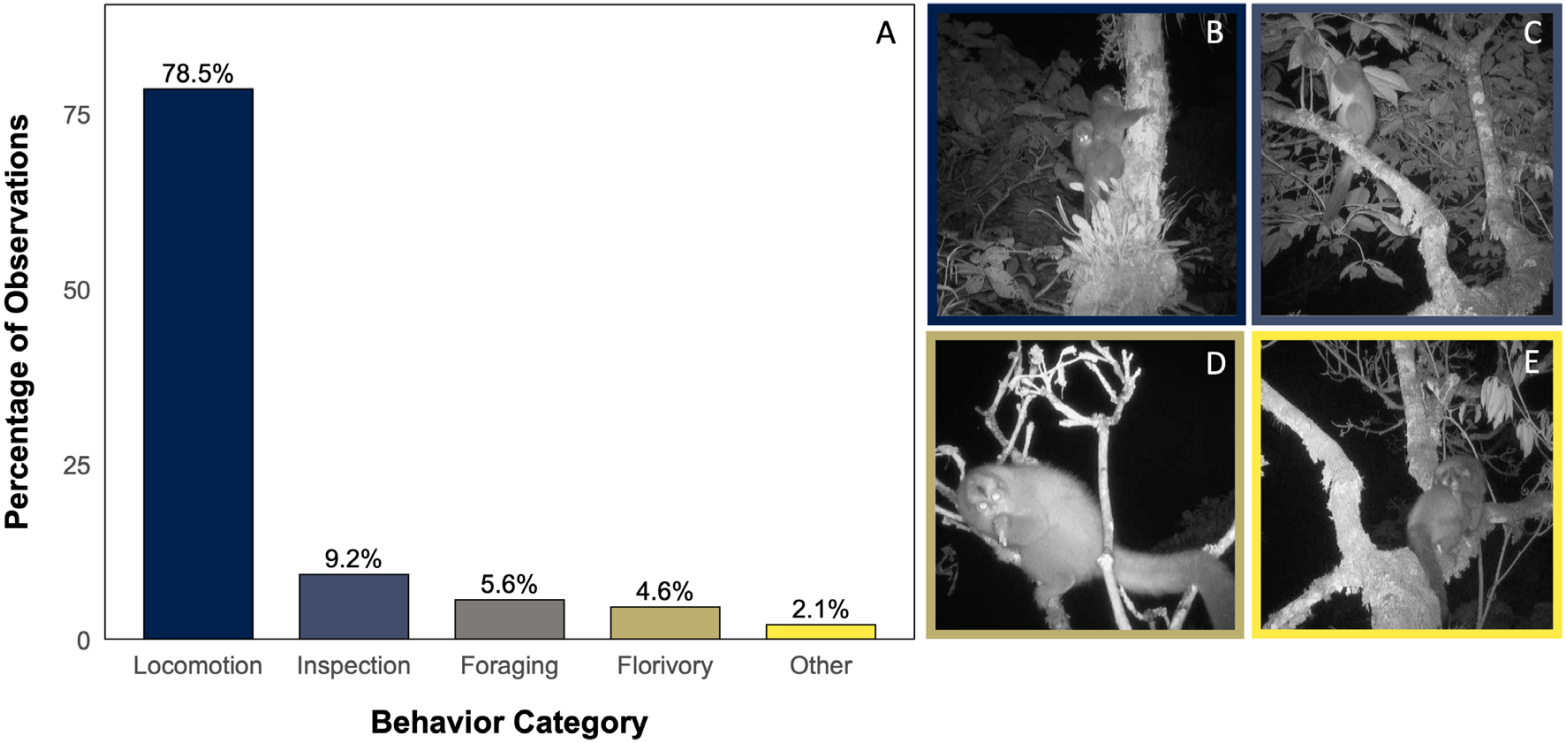
Behavioral observations of *Aotus vociferans* . (A) Percentage of observations per behavior category. Colors correspond to categories (dark blue = locomotion; gray‐blue = inspection; gray = foraging; gold = florivory; yellow = other). The pictures are camera‐trap images of selected behaviors: (B) locomotion: Adult climbing on a branch with an infant in dorsal clinging position; (C) inspection: Spix's night monkeys observing beneath the leaves of *Handroanthus chrysanthus* , likely searching for insects; (D) florivory: *A. vociferans* sucking the nectar at the base of a flower of *H. chrysanthus* ; (E) other: Social interaction between two individuals. “Foraging” lacks a photo due to difficulty capturing that action.

In most *A. vociferans* independent detections (78.50%; *n* = 153), we classified behavior (Figure 3; Table S4) as walking or jumping on the tree (i.e., locomotion), or looking for something edible (i.e., inspection, *n* = 18; 9.23%). In almost 5% of the events, we recorded direct consumption of flowers (Video 5) or nectar (i.e., florivory; *n* = 9; 4.62%). In a few cases, we also observed the monkeys apparently eating insects (i.e., insectivory; *n* = 8; 4.10%), leaves (i.e., herbivory, *n* = 2; 1.03%), and fruits' capsules (i.e., frugivory; *n* = 1; 0.51%) or socially interacting with other individuals (*n* = 2; 1.03%).

On three occasions, we recorded a group of five monkeys, the highest number of individuals detected together. In September 2023, we classified one of them as a young immature individual due to its more uncertain movements and smaller size (Video 6). On the two other occasions, in February and March 2024, we recorded four adults plus a fifth individual that was an infant. We first detected an infant on the 12th of January 2024, when it was carried by an adult in a ventral clinging position (Video 1). From the beginning of February to the 18th of April, when it made its last appearance, the infant seemed to be larger and was carried in a dorsal clinging position (Video 2). Social interactions with potential reproductive relevance were observed twice (12 and 16 July 2024, Videos 3 and 4), when two individuals engaged in apparent investigation of subcaudal secretion behavior, although no clear sexual activity was confirmed.

**VIDEO 1:** Adult Spix's night monkey ( *Aotus vociferans* ) carrying an infant in a ventral‐clinging position. The infant's tail is clearly visible between seconds 2 and 4. Video content can be viewed at https://onlinelibrary.wiley.com/doi/10.1002/ece3.73918.

**VIDEO 2:** Adult Spix's night monkey ( *Aotus vociferans* ) carrying an infant in dorsal‐clinging position. Video content can be viewed at https://onlinelibrary.wiley.com/doi/10.1002/ece3.73918.

**VIDEO 3:** Two adult Spix's night monkeys ( *Aotus vociferans* ) engaged in apparent mutual genital sniffing. Video content can be viewed at https://onlinelibrary.wiley.com/doi/10.1002/ece3.73918.

**VIDEO 4:** Three adult Spix's night monkeys ( *Aotus vociferans* ), with two individuals engaged in social interaction of potential reproductive relevance, indicated by an apparent attempt of genital sniffing from second 19. Video content can be viewed at https://onlinelibrary.wiley.com/doi/10.1002/ece3.73918.

**VIDEO 5:** Adult Spix's night monkey ( *Aotus vociferans* ) collecting a flower of *Handroanthus chrysanthus* and squeezing it while bringing it to the mouth, likely to extract nectar from it. Video content can be viewed at https://onlinelibrary.wiley.com/doi/10.1002/ece3.73918.

**VIDEO 6:** Group of five Spix's night monkeys ( *Aotus vociferans* ) jumping between branches of *Handroanthus chrysanthus* . The fourth individual, smaller than the others and closely followed by an adult, is likely a young immature individual, given its size and less agile movements. Video content can be viewed at https://onlinelibrary.wiley.com/doi/10.1002/ece3.73918.

### 3.3 Nocturnal and Seasonal Activity Pattern

**FIGURE 4:**
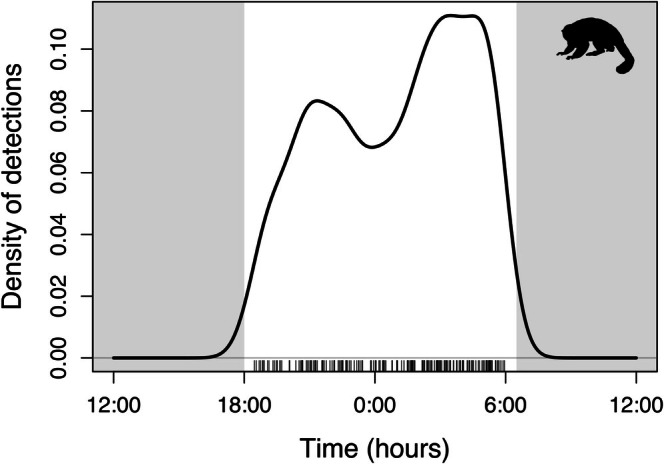
Nocturnal activity pattern of *Aotus vociferans* . Temporal distribution of detections across the 12.5‐h camera monitoring period (18:00–06:30) in the white area. The solid line shows the estimated activity density, with each tick mark representing one independent detection (*n* = 195). Shaded gray areas indicate daytime hours.

The nocturnal activity exhibited a bimodal pattern, with two distinct peaks: one at 22:00 and another at 05:00 (Figure 4). Over the 16‐month study period, from August 2023 to November 2024, *A. vociferans* detections averaged 12.19 ± 6.61 per month (range 3.00–22.00, Table S5), reaching a peak in May and July 2024. The peak of activity did not match the flowering peak of *H. chrysanthus* in August (flower buds) and September (open flowers). During the flowering time, the mean class values calculated across all monitored trees did not exceed the phenological class of 4 (Table S5). Total monthly precipitation averaged 119.29 ± 35.63 mm (range 55.10–162.90, Table S5), with higher rainfall values between December 2023 and April 2024.

### 3.4 *A. vociferans* Activity Pattern in the Context of Flower Availability and Seasonality Effects

We found no strong pairwise associations among the covariates included in our model (DBH‐weighted flower buds, PCP, sinM, and cosM), as all Spearman absolute correlation coefficients (|*ρ*|) were < 0.8 (Table S6). Variance inflation factor (VIF) values were < 5 (DBH‐weighted flower buds = 3.69, PCP = 1.66, sinM = 2.87, cosM = 2.59), indicating low multicollinearity among predictors (James et al. 2013). Thus, we included all of them as fixed effects in the negative‐binomial generalized linear mixed models (NB‐GLMMs).

**FIGURE 5:**
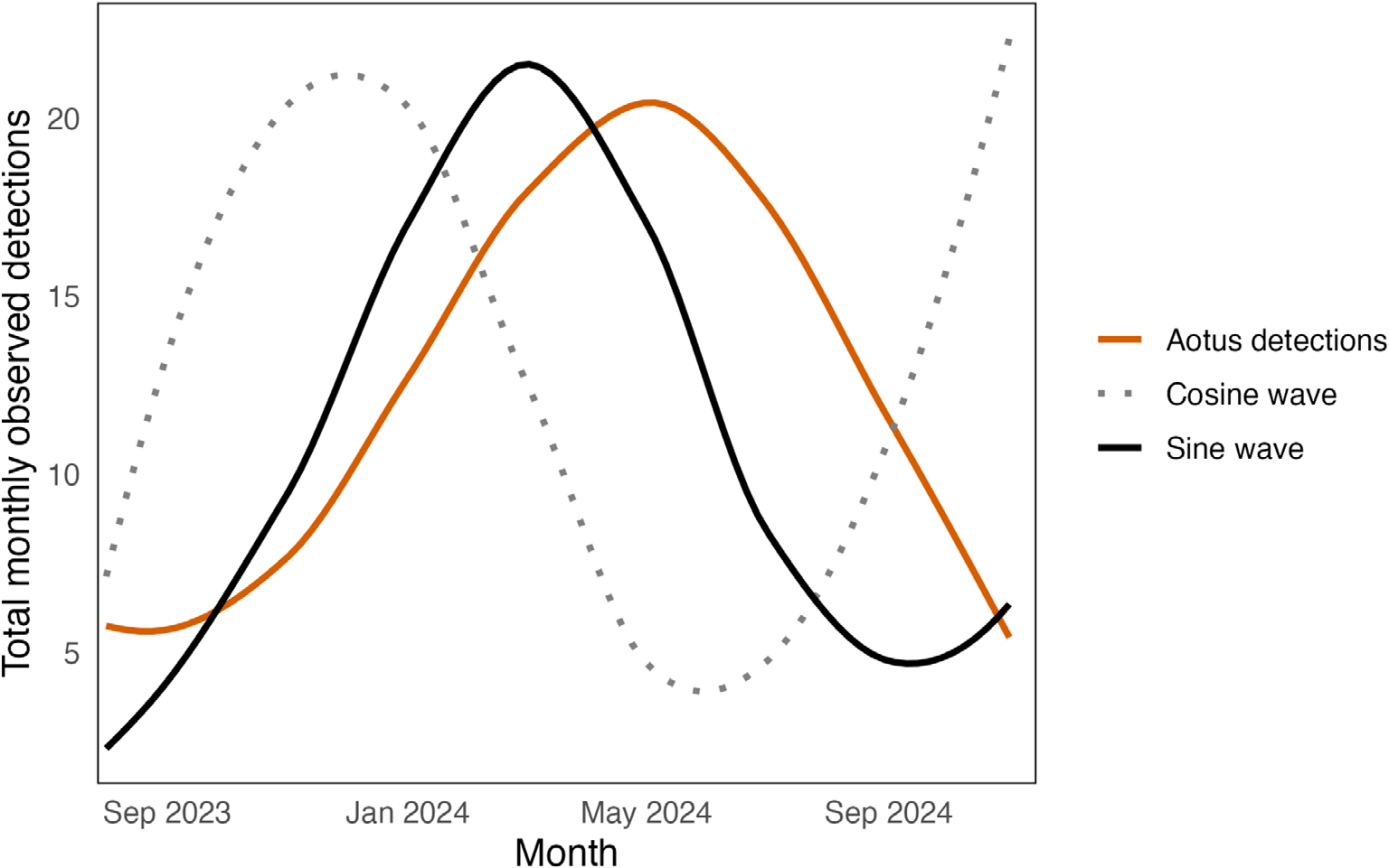
Observed total monthly detections of *Aotus vociferans* (orange; LOESS‐smoothed) compared with the sine (black, solid) and cosine (gray, dotted) seasonal functions used as circular predictors in the negative‐binomial GLMM. Only the sine term was retained as a significant predictor in the model. Sine and cosine waves were rescaled to the range of observed detections for visualization purposes.

The NB‐GLMM examining variation in the number of independent detections of *A. vociferans* per tree per month revealed substantial model‐selection uncertainty, with several models receiving similar support (Table S7). The random intercept (i.e., tree) in the top‐ranked model showed substantial variation in baseline detection rates among trees (variance = 0.96). However, since no single model was clearly supported, inference was based on model averaging across the confidence set of models with ΔAICc ≤ 2. Results from model averaging indicated that the circular seasonal variable (sinM) emerged as the strongest predictor (estimate = 0.76 ± 0.28 SE, *p* = 0.008; Table 3), reflecting the overall rise from autumn to mid‐year in the data (Figure 5). In contrast, DBH‐weighted flower buds (FB_DBH), precipitation (PCP), and the other seasonal variable (cosM) showed weak support, with model‐averaged coefficients close to zero and confidence intervals overlapping zero.

**TABLE 3:** Model‐averaged parameter estimates from negative‐binomial generalized linear mixed model (NB‐GLMM) explaining the detections of *Aotus vociferans* at tree per month level.

Predictor	Estimate	SE	*p*	Rate_ratio	CI_low	CI_high
Intercept	0.963	0.794	0.225	2.620	0.553	12.415
FB_DBH	0.010	0.020	0.626	1.010	0.971	1.050
PCP	−0.004	0.005	0.460	0.996	0.986	1.006
**sinM**	**0.756**	**0.283**	**0.008**	**2.130**	**1.222**	**3.711**
cosM	−0.086	0.166	0.605	0.918	0.662	1.271

*Note:* Estimates were averaged across the candidate model set with ΔAICc ≤ 2 using Akaike weights. The table reports the intercept, DBH‐weighted flower buds (FB_DBH), precipitation (PCP), and the circular variables for annual seasonality (sinM and cosM). Results are expressed as model‐averaged estimates, adjusted standard errors (SE), *p*‐values, rate ratios (RR), and Wald 95% confidence intervals (CI_low and CI_high). In bold, the strongest predictor (**sinM**) is highlighted.

Although the random intercept for tree ID indicated substantial among‐tree heterogeneity in detections (variance = 0.96, SD = 0.79), DHARMa diagnostics (Figure S2) of the top‐ranked model showed no evidence of overdispersion (*p* = 0.94), zero‐inflation (*p* = 0.95) or deviations from residual uniformity (*p* = 0.89).

## 4 Discussion

Despite the elusive nature of night monkeys, the arboreal camera traps deployed in this study recorded them frequently, averaging 12 detections per month. Although we could not distinguish individual animals, we believe that these detections correspond to a single group, given that the area covered by this study (0.12 ha) is much smaller than the home ranges reported for other *Aotus* species, which typically range between 4 and 10 ha (Fernandez‐Duque et al. 2008; van der Heide et al. 2012; Wright 1989, 1996).

The detections revealed a bimodal activity pattern during the night which, although obtained with different methods and from different *Aotus* populations and species, is consistent with previous studies (Link et al. 2023). However, unlike observations from an Amazonian population of *A. vociferans* (Fernandez‐Duque 2011), where activity peaked at dusk (between 17:50 and 19:30), the first peak in our study occurred later in the night (at 22:00). The second and highest peak occurred before sunrise (05:00), and activity declined during the central hours of the night, a behavior that was previously reported for *Aotus azarae* in Brazil (Garcia and Braza 1987). In some *Aotus* populations, cathemeral behavior was observed and linked to strong seasonal variation in light and temperature (Rotundo et al. 2000; Wright 1985). However, since our study was carried out in a tropical mountain rainforest at equatorial latitudes, where such variation is minimal, we consider the group we observed to be strictly nocturnal. Nevertheless, as cathemeral activity was also reported for rainforest populations of *Aotus nigriceps* (Khimji and Donati 2014), further studies including daytime sampling would be useful to confirm this pattern.

During their nocturnal activity, the observed *A. vociferans* individuals consumed flowers, fruits, and insects, confirming their dietary flexibility (van der Heide et al. 2023). Despite their clear attraction to *H. chrysanthus* flowers, the brief flowering pulse did not coincide with an increase in *A. vociferans* detections, suggesting that flowers and flower buds of this tree species only function as a complement to a much broader diet. Although we did not quantify the flowering and fruiting phenology of the surrounding plants, studies from the same area report that multiple tree species bear flowers (Bendix et al. 2006; Cueva‐Ortiz et al. 2006) and fleshy fruits throughout the whole year (Acosta‐Rojas et al. 2021; Quitián et al. 2019). This suggests a relatively continuous availability of fruit resources in the perhumid tropical mountain rainforest (Bendix et al. 2006; Cueva‐Ortiz et al. 2006), albeit provided by different plant species. Such patterns are typical of forests lacking a pronounced dry season, where fruiting peaks are less synchronized (van Schaik et al. 1993). Since the foraging preferences of *A. vociferans* at this site remain unknown, we cannot exclude the possibility that the phenology of specific preferred plant species might influence its seasonal activity pattern, as well as its dietary preferences. During periods when preferred foods are scarce, individuals may increase their consumption of flowers, as reported for several primates worldwide (Dammhahn and Kappeler 2008; Heymann 2011; Hogan et al. 2016) and for other *Aotus* species (Montilla et al. 2021; Wright 1989, 1996). Whether flower consumption reflects compensation for preferred food scarcity or is instead linked to reproductive timing, as suggested for *Alouatta palliata mexicana* (Gisbrecht et al. 2025), remains unclear.

The substantial variability among trees (random intercept variance = 0.96) indicates that unmeasured tree‐level factors may play an important role in shaping detection patterns. This highlights the potential influence of tree‐specific characteristics and suggests that increasing the number of sampled trees and associated covariates (e.g., their position accounting for surrounding flowering and fruiting tree species) could help to better capture this variability. Overall, the averaged models reported a strong effect of the circular variable for annual seasonality (sinM) indicating that *A. vociferans* detections followed a structured seasonal pattern, with a pronounced activity peak between May and July.

One plausible explanation is that this pattern is influenced by the reproductive cycle of the *A. vociferans* group observed in our study area, as suggested by the timing of infant detections by our arboreal camera traps in combination with information available in the literature.

Infants are typically carried in a ventral clinging position only during the first three to six weeks of their life (Dixson and Fleming 1981). Since we recorded an infant being carried in this position only in January 2024, we suggest that the infant's mother likely gave birth around November–December 2023. This timing is broadly consistent with observations from other *Aotus* species in the wild, including *Aotus trivirgatus* in Peru (Wright 1985) and *Aotus azarae* in Argentina (Fernandez‐Duque et al. 2002), where births were reported between August and January, and October and January, respectively. Assuming a similar reproductive schedule for the *A. vociferans* group in our study, the decline in detections during October 2023 could reflect reduced adult movement associated with the birth season. Although detailed long‐term reproductive data remain limited for most *Aotus* species, available observations across several populations suggest that births tend to occur mainly between October and March (Fernandez‐Duque et al. 2002; Fernandez‐Duque 2011; Wright 1985).

From February to April 2024, the infant was observed clinging dorsally. Although dorsal clinging can be observed as early as the first day of life, the transition from ventral to dorsal clinging generally corresponds to a later developmental stage, becoming more frequent from the third to fourth week (Dixson 1983), and lasting for a couple of months (Dixson and Fleming 1981). By April, the infant's size approached that of the adults, suggesting they would soon move independently as fathers would start pushing juveniles off their backs (Wright 1990). This developmental timeline coincides with the period during which adult females may become receptive again: in *Aotus azarae* , for instance, most conceptions are inferred to take place from May to September (Fernandez‐Duque et al. 2002).

Although *Aotus azarae* is the only species from the same genus for which detailed wild reproductive data exist, similar seasonal patterns have been anecdotally reported for other *Aotus* species (Corley et al. 2023). In our study, independent detections increased from May to July, suggesting elevated adult activity during this period. One possible explanation is that this increase reflects greater social interactions associated with the assumed reproductive phase. However, it could also result from increased freedom of movement following the release from direct parental care of a highly dependent infant. Consequently, although formal data for *A. vociferans* are lacking, we can hypothesize that mating could be concentrated within a restricted part of the year even in the group we observed.

The two videos recording an apparent investigation of subcaudal secretions observed in July further suggest the presence of social behaviors that may contribute to increased activity during these months, possibly linked to reproduction. However, given the limited number of studies available (Dixson 1983), the extent to which *Aotus* species in the wild exhibit strong reproductive seasonality remains unclear. In addition, although the increase in detections we observed between May and July is consistent with a possible mating phase of the Spix's night monkey group, we emphasize that our data do not guarantee the existence of a direct link between the observed activity pattern and the suggested reproductive cycle. As a consequence, the interpretation we present here should be regarded as a plausible hypothesis rather than a proven mechanism.

## 5 Conclusion

In this study, arboreal camera traps have proven to be a very useful tool for elucidating the natural history of *A. vociferans*, a small nocturnal and arboreal neotropical primate. Although our results are based on a limited number of replicates (trees and cameras) due to logistical limitations of tree‐climbing fieldwork, they provide biologically relevant insights into the ecology of *A. vociferans* . The seasonal activity pattern, together with the infant observations, provides preliminary evidence that may contribute to inferring aspects of the annual reproductive cycle of a group of night monkeys in a tropical mountain rainforest in Ecuador. The flower consumption and foraging data are important for a better understanding of the feeding behavior and ecology of *Aotus* species, which are understudied not only in Ecuador but also compared with other neotropical primate species (Fernandez‐Duque 2023).

Our results contribute to the understanding of the activity, reproductive biology, and diet of a nocturnal arboreal species and will hopefully stimulate similar research in Ecuador and in the canopies of other tropical forests.

## Author Contributions

**Claudia Viganò:** conceptualization (equal), data curation (lead), formal analysis (lead), investigation (lead), methodology (lead), project administration (equal), software (lead), visualization (lead), writing – original draft (lead), writing – review and editing (equal). **Alessandro Mainardi:** conceptualization (supporting), data curation (supporting), investigation (supporting), methodology (supporting), project administration (equal), writing – review and editing (equal). **Pedro Alonso‐Alonso:** conceptualization (supporting), writing – original draft (supporting), writing – review and editing (equal). **Malika Gottstein:** conceptualization (supporting), writing – original draft (supporting), writing – review and editing (equal). **Víctor Romero:** investigation (supporting), resources (supporting), writing – review and editing (equal). **Richar Samaniego:** investigation (supporting), writing – review and editing (equal). **Juan Pablo Uchuari:** investigation (supporting), writing – review and editing (equal). **Katrin Heer:** conceptualization (equal), funding acquisition (lead), project administration (equal), resources (lead), supervision (lead), writing – original draft (supporting), writing – review and editing (equal).

## Funding

The study was conducted in the frame of the Emmy Noether project “Phenology of tropical tree species—environmental cues, molecular mechanisms, and consequences for plant‐animal interactions” (HE 7345/8‐1) funded by the German Research Foundation (DFG). KH and MG were also supported by the Eva Mayr‐Stihl Foundation. MG was supported by the Heinrich Böll Foundation. VR was supported by UTPL through the project “Fortalecimiento a la gestión del Abra de Zamora, un área clave para la conservación de la biodiversidad y los servicios ambientales de los Andes del sur del Ecuador” (Project code: PROY_INV_BA_2022_3573).

## Ethics Statement

This research was conducted in full compliance with ethical standards for biological research under the research permit MAATE‐DBI‐CM‐2022‐0248.

## Conflicts of Interest

The authors declare no conflicts of interest.

## Supporting information


**Table S1:** Cameras height, trees coordinates and size.
**Figure S1:** Cameras' activity period.
**Table S2:** Trees distance matrix.
**Table S3:** Cameras' recording periods.
**Table S4:** *Aotus* behavior.
**Table S5:** Ecological covariates' descriptive statistics.
**Table S6:** Pairwise Spearman correlations among model's covariates.
**Table S7:** Model selection.
**Figure S2:** DHARMa diagnostics' plots.

## Data Availability

The data and code that support the findings of this study are openly available in Zenodo at https://zenodo.org/records/20591043. The GitHub repository containing the project files is available at https://github.com/claudiavigan/Publication_Aotus (Release Aotus.r1.3).
