# Supplementary material for: Flower Feeding and Reproductive Timing in Spix's Night Monkeys (Aotus vociferans): Evidence From Arboreal Camera Traps
Source: Ecol Evol. 2026 Jul 3;16(7):e73918. doi: 10.1002/ece3.73918 (PMC13329687; doi:10.1002/ece3.73918)

### Supporting Information for:

### Flower feeding and reproductive timing in Spix’s night monkeys (*Aotus vociferans*): evidence from arboreal camera traps

#

**Table S1 – Cameras height, trees coordinates and size**

Geographic coordinates, diameter at breast height (DBH) and height of the *Handroanthus chrysanthus* trees monitored during the study. Longitude and latitude are reported in decimal degrees (WGS84), DBH in centimeters and tree and camera height in meters.

| **TreeID** | **Longitude** | **Latitude** | **Tree DBH (cm)** | **Tree Height (m)** | **Camera Height (m)** |
| --- | --- | --- | --- | --- | --- |
| H01 | -79.0703 | -3.97465 | 44.9 | 25 | 22 |
| H04 | -79.07017 | -3.97488 | 62.1 | 27 | 25 |
| H05 | -79.07022 | -3.9747 | 55.1 | 29 | 21 |
| H06 | -79.07001 | -3.9747 | 63.39 | 29 | 24 |
| H11 | -79.07084 | -3.97481 | 70.38 | 32 | 24 |

**Figure S1 – Cameras’ activity period**

Total period of activation of the cameras (labels on the y axis) mounted on the *Handranthus chrysantus* trees (coloured lines). The months on the x axis cover the whole study period (from August 2023 to November 2024), and the shaded grey area highlights the time when all cameras were recording simultaneously.

**
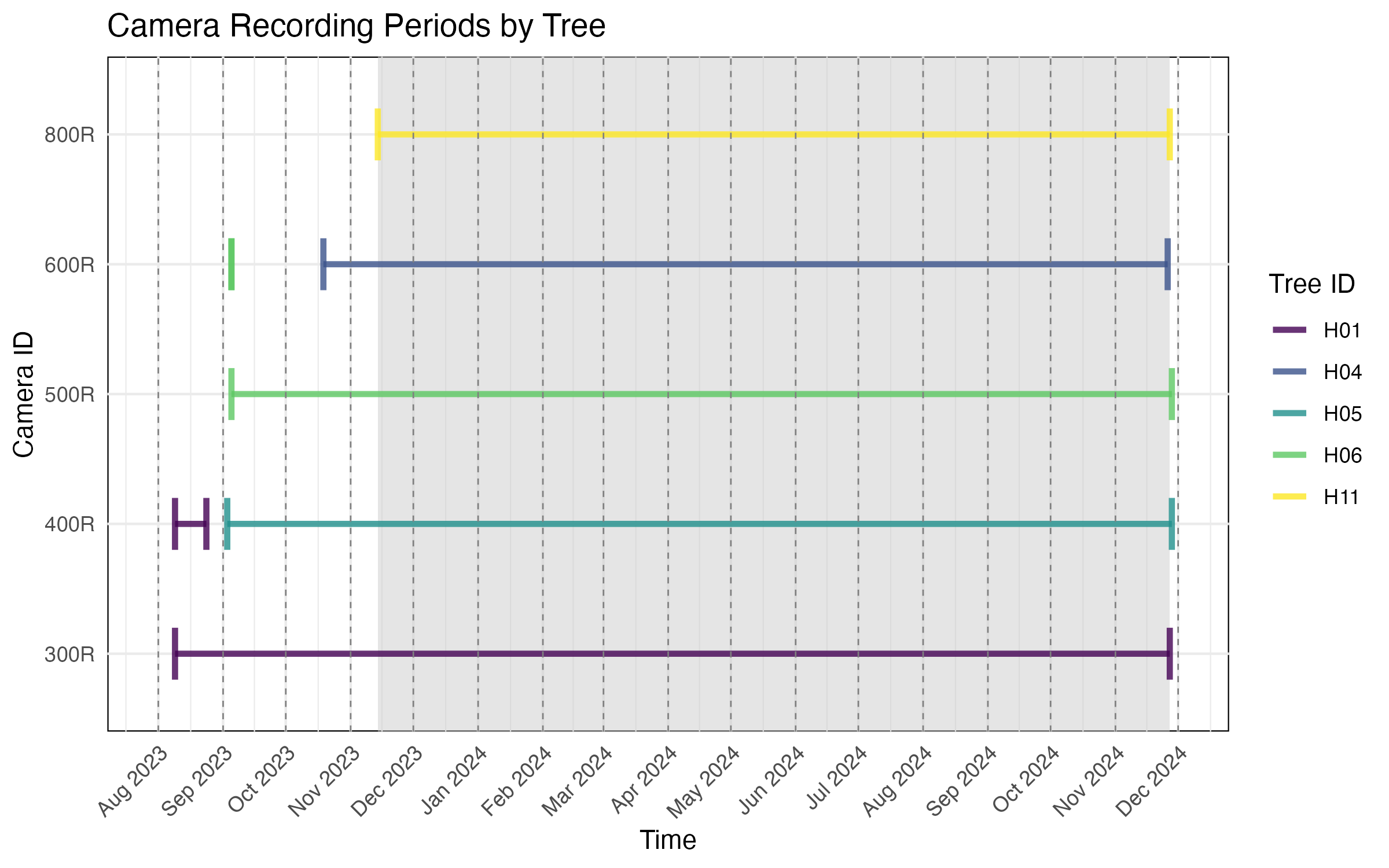
**

**Table S2 – Trees distance matrix**

Matrix of the total distance between each tree, and the mean distance of each tree from the others (Mean). Total mean distance is the average of the obtained mean distances. All measures are in meters.

| **Tree ID** | **H01** | **H04** | **H05** | **H06** | **H11** | **Mean** |
| --- | --- | --- | --- | --- | --- | --- |
| H01 | 0 | 29.4 | 10.5 | 32.7 | 62.6 | 33.8 |
| H04 | 29.4 | 0 | 20.8 | 26.8 | 74.8 | 37.95 |
| H05 | 10.5 | 20.8 | 0 | 23.3 | 69.9 | 31.12 |
| H06 | 32.7 | 26.8 | 23.3 | 0 | 93 | 43.95 |
| H11 | 62.6 | 74.8 | 69.9 | 93 | 0 | 75.08 |
| Total mean distance |  |  |  |  |  | 44.37 |

**Table S3 – Cameras’ recording periods**

The table lists, for each tree (*TreeID*) and camera (*CameraID*), the first (*StartDate*) and last (*EndDate*) day of camera activity. Camera trap nights (CTNs) represent the number of nights each camera was active for, calculated by summing the days within each camera’s activation period. The total number of CTNs is the sum across all cameras, whereas the total number of days reported (478) corresponds to the number of calendar days from the earliest start date to the latest end date.

| **CameraID** | **TreeID** | **StartDate** | **EndDate** | **CTNs** |
| --- | --- | --- | --- | --- |
| **300R** | H01 | 9-Aug-23 | 27-Nov-24 | 477 |
| **400R** | H01 | 9-Aug-23 | 24-Aug-23 | 16 |
| **400R** | H05 | 3-Sep-23 | 28-Nov-24 | 453 |
| **500R** | H06 | 5-Sep-23 | 28-Nov-24 | 451 |
| **600R** | H04 | 19-Oct-23 | 26-Nov-24 | 405 |
| **600R** | H06 | 5-Sep-23 | 5-Sep-23 | 1 |
| **800R** | H11 | 14-Nov-23 | 27-Nov-24 | 380 |
| **Total number of days** | | 478 | | 2,183 |

**Table S4 – *Aotus* behavior**

Number of detections (n) and their proportion (%) over the total, reporting the observed behaviors (in bold) and associated single actions (in italics). Behavior totals are obtained by summing the values of the corresponding actions. The sum of the behavior totals corresponds to the total number of independent detections (n=195) of *Aotus vociferans* obtained in this study.

| **Behavior** | **n** | **%** |
| --- | --- | --- |
| **Locomotion** | 153 | 78.5 |
| **Inspection** | 18 | 9.23 |
| **Foraging** | 11 | 5.64 |
| *Insectivory* | *8* | *4.1* |
| *Herbivory* | *2* | *1.03* |
| *Frugivory* | *1* | *0.51* |
| **Florivory** | 9 | 4.62 |
| *Florivory* | *7* | *3.59* |
| *Nectarivory* | *2* | *1.03* |
| **Other** | 4 | 2.05 |
| *ND* | *2* | *1.03* |
| *Social* | *2* | *1.03* |

**Table S5 – Ecological covariates’ descriptive statistics**

Summary statistics for the ecological covariates included in the study. The monthly activity pattern of *Aotus* *vociferans* (“Aotus detections”) was treated as a discrete numerical variable (count of independent detections per month). Flowering phenology covariates (“Flower buds” and “Open flowers”) were derived from ordinal phenological classes (Table 1). For each month, we calculated the mean phenological class across all monitored trees; therefore, the reported means and standard deviations describe the distribution of these monthly class scores rather than precise counts of flower buds or flowers . Rainfall (“Total monthly precipitation”) was treated as a continuous numerical variable (mm). Units are shown in parentheses.

| **Variable** | **Mean** | **Median** | **SD** | **Min** | **Max** |
| --- | --- | --- | --- | --- | --- |
| Aotus detections (count/month) | 12.19 | 13.00 | 6.61 | 3.00 | 22.00 |
| Flower buds (mean phenological class/month) | 1.42 | 0.25 | 1.73 | 0.00 | 4.00 |
| Open flowers (mean phenological class/month) | 0.56 | 0.00 | 1.07 | 0.00 | 3.43 |
| Precipitation (mm/month) | 119.29 | 117.15 | 35.63 | 55.10 | 162.90 |

**Table S6 – Pairwise Spearman correlations among model’s covariates**

Pairwise Spearman’s rank correlation coefficients (ρ) among the selected covariates for flowering phenology (DBH-weighted flower buds: FB_DBH), rainfall (total monthly precipitation: PCP), and annual seasonality (circular covariates: sinM and cosM).

| **Variable 1** | **Variable 2** | **Spearman** ρ |
| --- | --- | --- |
| cosM | FB_DBH | -0.69 |
| sinM | PCP | 0.60 |
| sinM | FB_DBH | -0.50 |
| PCP | FB_DBH | -0.47 |
| cosM | PCP | 0.21 |
| cosM | sinM | -0.01 |

**Table S7 – Model selection**

Model selection results for negative binomial generalised linear mixed models (NB-GLMMs) of *Aotus vociferans* independent detections per tree per month. Candidate models included all possible subsets of the fixed effects DBH-weighted flower buds (FB_DBH), total monthly precipitation (PCP), and annual seasonality (sinM and cosM). Models were ranked by corrected Akaike Information Criterion (AICc). For each candidate model, the number of parameters (df), log-likelihood (logLik), AICc, ΔAICc (difference from the top-ranked model), and Akaike weight (weight) are reported. Models with ΔAICc ≤ 2 were considered part of the confidence set used for model averaging.

| **Model_terms** | **df** | **logLik** | **AICc** | **DeltaAICc** | **weight** |
| --- | --- | --- | --- | --- | --- |
| PCP + sinM | 5 | -147.67 | 306.14 | 0 | 0.180 |
| sinM | 4 | -148.97 | 306.47 | 0.33 | 0.153 |
| cosM + sinM | 5 | -147.84 | 306.49 | 0.35 | 0.152 |
| FB_DBH + PCP + sinM | 6 | -146.78 | 306.71 | 0.57 | 0.135 |
| cosM + PCP + sinM | 6 | -146.86 | 306.86 | 0.72 | 0.126 |
| FB_DBH + sinM | 5 | -148.04 | 306.89 | 0.75 | 0.124 |
| cosM + FB_DBH + sinM | 6 | -147.79 | 308.72 | 2.58 | 0.050 |
| cosM + FB_DBH + PCP + sinM | 7 | -146.71 | 308.98 | 2.84 | 0.044 |
| cosM + FB_DBH | 5 | -150.22 | 311.24 | 5.10 | 0.014 |
| (Intercept-only) | 3 | -153.38 | 313.08 | 6.94 | 0.006 |
| cosM | 4 | -152.45 | 313.44 | 7.30 | 0.005 |
| cosM + FB_DBH + PCP | 6 | -150.21 | 313.58 | 7.43 | 0.004 |
| FB_DBH | 4 | -153.06 | 314.65 | 8.51 | 0.003 |
| PCP | 4 | -153.07 | 314.67 | 8.53 | 0.003 |
| cosM + PCP | 5 | -151.95 | 314.71 | 8.57 | 0.002 |
| FB_DBH + PCP | 5 | -152.93 | 316.68 | 10.54 | 0.001 |

**Figure S2 – DHARMa diagnostics’ plots**

DHARMa simulation-based residual diagnostics for the best-fitting negative binomial GLMM. (Left) Quantile-quantile plot of scaled residuals indicates no deviation from the expected uniform distribution (Kolmogorov–Smirnov test: p = 0.89), with no evidence of overdispersion (p = 0.94) or outliers (p = 1). (Right) Residuals versus rank-transformed predictions show no systematic structure, confirming adequate model fit.


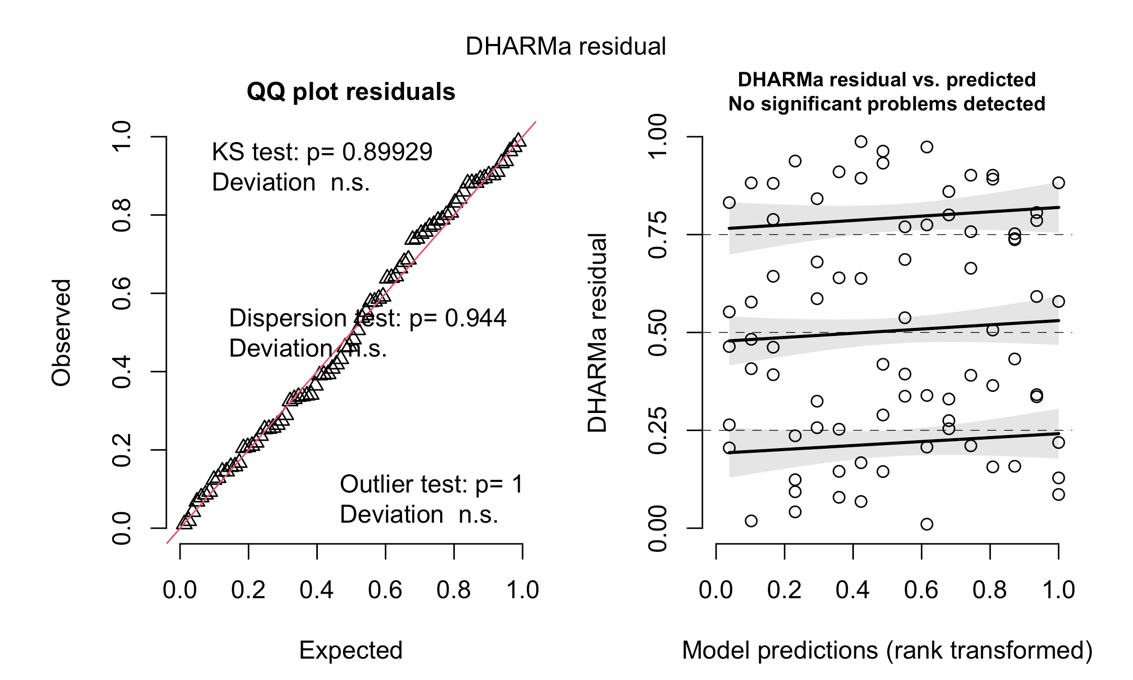

Supplement: Supplementary file 7 — Table S1: Cameras height, trees coordinates and size. Figure S1: Cameras' activity period. Table S2: Trees distance matrix. Table S3: Cameras' recording periods. Table S4: Aotus behavior. Table S5: Ecological covariates' descriptive statistics. Table S6: Pairwise Spearman correlations among model's covariates. Table S7: Model selection. Figure S2: DHARMa diagnostics' plots. [file ECE3-16-e73918-s001.docx]
